# Understanding contraceptive choice after discontinuing the contraceptive pill in Germany - a qualitative inquiry

**DOI:** 10.1186/s12978-026-02416-8

**Published:** 2026-07-23

**Authors:** Jana Niemann, Lisa Glaum, Liane Schenk

**Affiliations:** 1 Institute of Medical Sociology (IMS), Interdisciplinary Center for Health Sciences, Medical School of Martin, Luther University Halle-Wittenberg, Halle, Germany; 2 Institute for Medical Sociology and Rehabilitation Science, Charité - Universitätsmedizin Berlin, Berlin, Germany

**Keywords:** Discontinuation, Contraceptive pill, Contraception, Hormones, Pill, Contraceptive behavior

## Abstract

**Background:**

Contraception decision-making is a vital aspect of sexual and reproductive health, with individuals weighing various factors when choosing their current contraceptive methods. Method discontinuation is common and should not be viewed as an endpoint but rather as a starting point for selecting the next contraceptive option. However, there is a limited understanding of what former contraceptive pill users seek after discontinuation.

**Methods:**

This study examined the decision-making process and experiences of former oral contraceptive users (*N* = 19) who transitioned to alternative methods in Germany. We conducted a reflexive thematic analysis.

**Results:**

We generated five key themes shaping post-pill contraceptive decisions: (1) prioritization of efficacy and pregnancy prevention, with permanent methods offering psychological relief; (2) reliance on familiarity, particularly with condoms, as a default choice; (3) strong preference for hormone-free options due to concerns over side effects and bodily autonomy; (4) embodied aversion to invasive procedures, such as IUD insertion or complex surgeries; and (5) a growing emphasis on shared responsibility and gender equality in contraception, with calls for greater male involvement and accessible non-hormonal alternatives. These findings underscore the importance of individual experiences, bodily autonomy, and equitable contraceptive access in post-pill decision making.

**Conclusions:**

This study offers valuable insights into the complex nature of contraceptive decision-making, addressing considerations such as long-term health effects, bodily autonomy, and the need for improved contraceptive options.

## Abstract (plain language summary)

Choosing a birth control method is important for sexual and reproductive health. People consider many factors when selecting a method, and they often switch methods. However, we know little about what people want after they stop using birth control pills. In this study, we spoke to 19 former pill users in Germany. We wanted to understand how people choose birth control after stopping the pill. After stopping the birth control pill, many women wanted a method that worked well and helped prevent pregnancy, with some choosing long-term options, such as sterilization, for peace of mind. Others went back to what they already knew and felt comfortable with; most chose condoms because they were familiar and easy to use. The interviewees also wanted to avoid hormones, were worried about side effects or how birth control pills affected their bodies, and preferred natural or non-hormonal options. Some also felt uncomfortable with medical procedures like IUDs or surgeries, especially those that felt invasive or risky. At the same time, many wanted to share the responsibility of contraception with their partners. They hoped men would take more part, for example, by using condoms or getting vasectomies. Overall, people wanted choices that fit their bodies, lives, and relationships, and they wanted to feel in control of their health decisions. Our study showed that changing birth control methods is complex and personal. It highlights the need for better support, more choices, and attention to long-term health and personal preferences in birth control.

## Background

Contraceptive decision-making is a dynamic process that extends over the entire sexual and reproductive lifespan [1]. This underscores the need for comprehensive, scientifically substantiated, and unbiased sexual education and contraceptive counseling throughout life, empowering individuals seeking contraceptive counseling to navigate the complex landscape of contraceptive choices. Senderowicz (2019) described how distorted advice, fear tactics, and false or incomplete medical information can lead women^1^ to not use their preferred contraceptive method [2]. In addition, it can also impact the relationship between individuals seeking contraception and their medical provider [3, 4]. A growing loss of trust can lead to women seeking knowledge elsewhere and making fewer visits to healthcare providers [5]. Owing to provider bias, the advantages of hormone-based contraception and long-acting reversible contraceptives are described and prescribed more frequently [4]. This influence reproduces normative ideas and can lead women to choose contraceptive methods that do not meet their needs [6, 7].

Scholars have also shown that women who use oral contraceptives feel a stronger urge to take responsibility for contraception [8, 9]. This phenomenon raises questions regarding contraceptive choices and responsibilities when women discontinue oral contraceptive use.

Research on the discontinuation of oral contraceptives has shown that side effects are a predominant reason for method discontinuation [10–12]. Le Guen et al. [13]. summarized in a systematic review, that the reason for the discontinuation of hormonal contraception in Western countries is often related to side effects, psychological effects, and fertility fears. There is also a desire for nonhormonal contraception [13]. Furthermore, Thomé (2024) summarized the following: “They are not asking for less contraception, but for contraception that is better suited to their needs” [14].

However, these lived preferences and experiences in contraceptive decision-making are currently overlooked in (post-)discontinuation research [15, 16], leading to a gap in understanding this transitional phase. This study therefore aims to explore the decisions and wishes of 19 former contraceptive pill users in Germany.

## Data and methods

This study is part of a broader project examining the experiences of individuals who (dis)continued contraceptive pills in Germany [17]. We conducted in-depth interviews with former users, which adopted a biographical approach to explore the personal and practical factors influencing their (dis)continuation [18] and their lived experiences in contraceptive counseling [19]. In this publication, we focus on the aspects of interviews with former users that delve into the personal and practical aspects of contraceptive method selection after discontinuation of the contraceptive pill.

This study adhered to the ethical standards outlined in the current version of the Declaration of Helsinki. This study was approved by the Medical Faculty Ethics Committee of Martin Luther University Halle-Wittenberg (Reg-Nr. 2021-034). Informed consent was obtained from all participants.

### Study context

In Germany, there is no differentiation between the fields of gynecology, obstetric care, and family planning; these responsibilities are collectively managed by outpatient gynecologists. To qualify as an outpatient gynecologist, one must first complete clinical training [20]. Since 2007, the use of oral contraceptives in Germany has decreased from 55% to 38%, whereas condom usage has increased to 53%, making it the most prevalent method. This trend is particularly notable among young women, with only 22% of those under 22 years of age being prescribed the pill in 2022, a significant drop from 43% in 2015 [21]. Vieth et al. [3] highlighted that young German women (aged 18–29) often hold less positive views of oral contraceptives and have lower trust in gynecologists when they rely on informal sources such as friends, family, or the Internet for information. This reliance on informal channels may contribute to the widespread discontinuation of oral contraceptive pills, which is frequently discussed in the (social) media [22, 23]. However, Vieth et al. [3] also emphasized the strong desire among these women for accurate, accessible education about side effects and contraceptive options.

### Design and participants

Our analysis, grounded in critical realism and constructionist epistemology, situates interviewees’ lived experiences within broader social, cultural, and political contexts, recognizing reality as shaped by discourse and using researcher subjectivity as a valuable tool in knowledge generation [24, 25]. From December 2022 to September 2023, JN conducted 19 semistructured episodic interviews with former contraceptive pill users. The participants were German-speaking, aged 19–38, and included cisgender women and one nonbinary person. All lived in Germany during the study period, with one exception: one had moved overseas but maintained in contact with a German gynecologist. One interviewee from Italy relocated to Germany and switched to German healthcare providers. The participants’ education ranged from secondary school qualifications to master’s degrees, with most reporting good or excellent health. One had children, and the other was expected, with at least a six-month gap between stopping contraceptive pill use and conception; the average time since the last discontinuation of the oral contraceptive was six months. More than half of the participants had started using contraceptive pills multiple times.

### Data collection

All the interviews were conducted online. JN recruited former users through social media and her wider social networks. Interested individuals contacted the JN via email, and upon confirming eligibility, they received the study details, consent forms, and data protection information. JN offered a phone call to address questions and arrange interviews with those who agreed to participate. The topics in the interviews with former pill users included the participants’ relationships with the contracetive pill, initial use, gynecologist consultations, usage experiences, discontinuation processes, postdiscontinuation experiences, and evolving attitudes toward hormonal contraception [18]. In this analysis, only postdiscontinuation experiences evolving during the decision-making process for the postdiscontinuation method were included. JN conducted two pilot interviews before the data collection for both interview groups. We then refined the interview guides. For former pill users, data collection was continued until thematic saturation was achieved. 

### Data generation

We used a reflexive thematic analysis approach [25, 26] to develop themes informed by the contraceptive journey theory [1]. JN and LG read the transcripts thoroughly and identified recurring themes and patterns related to the research topics. Using MAXQDA, they actively coded the data and held weekly meetings to refine it. JN organized the semantic codes into preliminary themes that were evaluated by the research team. JN engaged in an iterative process of reviewing and developing themes, reorganizing them, and creating a thematic map. The research team critically examined the definitions, meanings, and interpretations of the themes. We also ensured intersubjective validation through collaborative analysis within the author team and presented the theme development in a qualitative interpretation circle at one university.

### Positionality and reflexivility

In the following section, we describe our reflexivity on this article’s topic and our position as researchers in qualitative health research. In particular, JN and LG repeatedly reflected on their views concerning the data while conducting and analyzing the research. The research team agreed that equitable access to modern contraception is a fundamental sexual and reproductive right.

#### JN’s positionality and reflexivity

As a cisgender woman with personal experience the contraceptive pill, discontinuation, and contraceptive responsibility in heterosexual relationships, I acknowledge the potential bias in my research. I reflected on how my experiences might influence my interpretation, especially regarding contraceptive choice and decision-making. To address this, I sought diverse perspectives within the data to ensure that the themes reflected collective narratives, not just my own. Regular discussions with LG and LS further helped to prevent any single perspective from dominating our interpretations.

#### LG’s positionality and reflexivity

I recognize that my research is influenced by my position. Through private discourse on oral contraceptive use, my thinking is also influenced by the opinions I encounter there. In my exchange with JN, I realize that I am familiar with some of these experiences that correspond with the data, and others that contradict it. Negotiating this through reflection and focusing on the data helps our interpretation to represent more than one perspective.

#### LS’s positionality and reflexivity

Identifies themselves as a living being of the human category, which is at home in empirical social research using both quantitative and qualitative methods. LS brought methodological reflexivity to the project and engaged in discussions with JN to ensure that the analysis remained grounded in the qualitative nature of the data and the participants’ lived experiences.

## Results

Individuals consider various factors when choosing their current contraceptive method. Overall, our results highlight the importance of individual experiences and preferences in selecting a suitable contraceptive method. After discontinuation, most participants used condoms (*n* = 10, 53%), followed by female sterilization (*n* = 3, 16%), vasectomy (*n* = 2, 11%), sympto-thermal method^2^ (German: *Natürliche Familienplanung)* (*n* = 1, 5%), hormonal rings (*n* = 1, 5%), progestogen coils (*n* = 1, 5%), and no contraceptive methods (*n* = 1, 5%). Through our inductive thematic analysis, we developed five key themes: (1) efficacy and pregnancy prevention, (2) seeking familiarity with previous contraception experiences, (3) pursuing hormone-free options, (4) embodied perceptions, and (5) shared responsibility and autonomy.

### Theme 1: efficacy & pregnancy prevention

Pregnancy prevention remains a crucial aspect of contraceptive decision-making after contraceptive pill discontinuation. For those who choose short-term contraception, such as condoms, the emphasis often lies on ‘known security’ (Sophia^3^) and familiarity with the method, especially compared to ‘taking the temperature’ (Lydia).

Those who have firmly decided against having children often opt for long-term contraceptive solutions such as vasectomy or sterilization. These more permanent methods provide a sense of security and mental relief, as exemplified by Luca’s experience with sterilization:

‘I was super happy. So, neither of us wants to have any more children or offspring. This security is very important for both of us and relaxes both of us mentally. We’ve already noticed that. And then just knowing: Okay, it’s almost impossible that I could somehow get pregnant.’.

The finality of these choices marks a significant endpoint in reproductive responsibility. It offers liberation from the constant concern of unintended pregnancies. However, the path is fraught with challenges, particularly for younger individuals who face significant barriers to accessing these procedures. Healthcare providers may question their decision-making capacity or impose arbitrary age restrictions, which are not only frustrating but also disempowering. Emma’s emotional journey to sterilize highlights the frustration and relief experienced by those who have long known that they do not want children but struggle to have their choices respected:

It’s been clear to me for over ten years now: I do not want to have children. That has never changed. But as a young woman, of course, it is incredibly difficult to face this option. […] I actually cried when I made the appointment because I never thought I would get this opportunity.

The societal implications of these barriers extend beyond personal struggles. Arbitrary age restrictions reflect systemic issues within healthcare, where policies often fail to account for individual circumstances or maturity.

### Theme 2: familiarity as a default in contraceptive decision-making

Our interviewees tended to opt for contraceptive methods they were already familiar with. This pattern indicates that familiarity significantly influences the decision-making process, not necessarily because they are actively seeking familiar methods, but because they default to what they know and find comfortable. Consequently, it is unsurprising that most of our interviewees chose condoms as their contraceptive method after the contraceptive pill was discontinued.

Some participants actively sought familiar contraceptive methods, with comfort and confidence from previous experience with condoms playing crucial roles in their decision-making. Sophia’s experience exemplifies this tendency, as she mentioned that her decision was shaped by her prior exposure to condoms. Her account suggests a reliance on what is known and trusted, underscoring the importance of familiarity in decision-making.

So what probably helped me decide is that yes/well, I have seen and used a condom before. […] So, yeah, a system that works, so to speak, helped a lot.

Others, however, had to seek the familiar condom because the other options were not satisfactory. For instance, Fannie expressed:

Condoms and that’s it. It’s also a bit of a topic because I… It’s okay. It’s not super bad. (…) But it was better with the pill because it’s more spontaneous. You don’t have to think about the condom. But the alternatives still don’t convince me. (laughs) […] So yes, it’s condoms.

This decision underscores the complexities of contraceptive decision-making, where systemic issues undermine personal preferences. This emphasizes the need for more satisfying contraceptive options that better align with personal needs and preferences.

### Theme 3: pursuing hormone-free contraceptive options

The aversion to hormonal birth control methods is a prominent theme among former users, with many expressing a strong preference for nonhormonal alternatives. This shift in attitudes stems from concerns regarding the potential side effects and long-term health implications of hormonal contraceptives, as well as the lived experiences with the contraceptive pill. The participants cited options such as condoms and sterilization as preferable alternatives and viewed them as safer (regarding their health impact) and more natural choices for managing their reproductive health. This sentiment was particularly evident in Natalia’s statement:

‘There are no more hormones, no more hormone injections, no more hormone patches, I do not want to know about that anymore’.

This antihormone stance, rooted in their vernacular knowledge (a form of knowledge rooted in veryday life, bodily experience, and social exchange) [15], appears to be ingrained in former users’ perspectives on birth control. Negative associations with hormonal methods, particularly the contraceptive pill, were consistently expressed throughout the interviews. Julie’s desire to ‘get off the hormones’ encapsulates a broader trend among respondents, suggesting a growing disillusionment with hormonal contraceptives. This indicates that after discontinuation, former users increasingly seeking methods that align with their concerns about bodily autonomy and long-term health impacts. Therefore, for most interviewees, it was not an option to use other hormonal contraceptive methods.

At the end of the interview, the participants were asked what they wanted regarding contraception. Many expressed a strong preference for nonhormonal contraceptive options, perceiving them as safer (regarding their health impact) and more natural alternatives to hormonal methods.:

‘What would I wish for? Yes, a safe alternative, as safe as the pill, but as a natural contraceptive.’ (Heather).

In Heather’s account, alternative contraception equals the natural contraceptive option. This naturalness is perceived as nonhormonal, reflecting concerns regarding the potential side effects of hormonal contraceptives. The participants emphasized the importance of finding alternatives that offer comparable contraceptive efficacy to hormonal methods while being perceived as more natural and less invasive:

‘I hope that someday there will be a fairly simple long-term contraceptive option for women with some sort of minimally invasive procedure’ (Luca).

### Theme 4: embodied aversion of invasive procedures

In this theme, we demonstrate that contraceptive decision-making is not only a cognitive or logistical calculation. Former users considered other embodied concerns in their decision-making processes. Some expressed discomfort with the idea of an IUD, viewing it as a foreign object in their bodies. Fannie’s hesitation – ‘I could not imagine having an IUD inserted either’ –speaks to a deeper, embodied resistance: the discomfort of imagining a small, foreign object being placed within a space traditionally associated with intimacy, vulnerability, and selfhood.

Similarly, Luca highlighted how the fear of surgical procedures could influence contraceptive choices. Although sterilization is a surgical intervention, their preference for it, if it were as minimally invasive as male sterilization, indicates an embodied reaction to different contraceptive methods. Luca’s sentiment suggests a preference for a simpler sterilization procedure that feels more in harmony with the body. This underscores the impact of perceived procedural complexity on contraceptive decision making.

If I had the choice, or if the procedure were as simple as the man’s, I would have chosen sterilization right away.

### Theme 5: shared responsibility and autonomy

Our respondents’ experiences highlighted the importance of partner involvement and shared responsibility in contraceptive decision-making. Many participants emphasized the value of open communication and the joint consideration of risks and benefits. For instance, Heather described her process as a collaborative effort, stating,

’So we have already agreed that this is now our joint responsibility and my partner’s additional responsibility. And we’ve talked about the risks of not using condoms or using them responsibly.’

This approach fosters confidence in the decision-making process. Similarly, Rachel viewed her partner’s decision to have a vasectomy as a positive step toward equalizing contraceptive responsibility within their relationship: ‘I felt good about it because I felt that my partner was not just, in quotes, passing the whole issue of contraception on to me, but that he was somehow taking his share of the risk.’ While partner involvement was significant for many respondents, some emphasized autonomy in decision-making. These individuals reported making choices independently without feeling pressured by their partners.

In addition, they expressed a desire for greater gender equality in terms of contraception. Gender equality is often described in terms of shared responsibilities and contraceptive options. Some want men to take more responsibility for contraception beyond the use of condoms. There is also hope for greater investment in male contraceptive options and alternatives that involve men playing a more active role in contraception. In this way, the risks and responsibilities of contraception can be shared within a partnership:

‘A partner with whom I can practice all non-hormonal contraceptive methods. (…) Where the responsibility for contraception is not only with us women but on both sides (laughing).’ (Olive).

In Olive’s description, we can not only see this need for shared responsibility but also that she wants to find a partner with whom she can practice nonhormonal contraceptive options. Sharing responsibility for contraception requires a partner who is willing to take risks. This was echoed by Coral, who said, ‘I think, in general, I would like my partner to feel a little more responsible for it (laughs).’

After discontinuing use, former users demonstrated increased autonomy in their choice of contraceptive method—a withdrawal from medical authority based on a profound undermining of their subjective agency during their pill history. Olive’s statement reveals a fragile relationship with medical authority: ‘If I do not (…) go along with the doctors’ attempts, I will not get any support. (…) That was again/That was outside the discussion.’ Now, after discontinuing use, a latent but effective self-competence is unfolding – “vernacular knowledge” that establishes itself beyond medical discourse.

## Discussion

Our findings highlight the complex interplay of factors [1] influencing contraceptive choices and wishes after the discontinuation of the contraceptive pill, including efficacy, previous experiences, aversion to hormonal methods, physical and psychological concerns, partner involvement, desire for safety, and gender equality. Our findings relate to Victoria Newtons work [15], as she frames such knowledge as vernacular knowledge: the informal, everyday, body-centered, and culturally embedded understanding of health and contraception acquired through lived experience, social networks, and collective wisdom. Our research supports the need for healthcare professionals to incorporate and listen actively to the individual’s wishes regarding contraceptive choice after discontinuation [5, 28].

The discontinuation of the contraceptive pill can influence the distribution of contraceptive responsibility between partners, particularly when alternative methods such as condoms or vasectomy are selected. This underscores the significance of autonomy and shared responsibility within a partnership and highlights the impact of social norms on allocating responsibility [9, 29]. Interestingly, alignment of contraceptive methods with sex is associated with contraceptive responsibility. *Women* can only escape if they choose a contraceptive alternative that can be assigned to the biological male part [6, 9].

The rejection of hormonal contraceptive methods is largely driven by concerns regarding side effects and long-term health risks. Scholars have highlighted the critical role of hormones and the increasing demand for natural contraceptive alternatives [8, 13, 28, 30]. Otte et al. [8]. identified the cumulative attribution of side effects to the contraceptive pill as a significant factor contributing to method discontinuation. However, some researchers caution that informing patients about these side effects may provoke a “nocebo effect,” potentially increasing the incidence of adverse reactions [31, 32]. As a result, it has been suggested that adverse effects should not be emphasized during consultations [31]. A recent study [33] indicates that this concern remains prevalent in clinical practice, where healthcare providers may dismiss patient-reported side effects as a “reverse placebo effect” or misconceptions influenced by online misinformation. This dismissive stance raises ethical issues by undermining contraceptive autonomy and agency, compromising the quality of long-term care, invalidating patients’ experiences, and eroding trust [5, 33, 34]. For instance, Beumer et al. [28] described negative experiences with (hormonal) contraception, condoms, long-acting contraception, and contraceptive counselling as one of the major reasons and common themes for unexpected pregnancy in their mixed-methods analysis. Further, Algera [35] demonstrated that self-tracking enables users to validate their embodied experiences and side effects, reframing them as legitimate data rather than mere nocebo effects. This validation reinforces the decision to pursue natural contraceptive options.

Safety, familiarity, and naturalness are crucial for the selection of contraceptive methods. Consequently, most interviewees chose to use condoms after stopping the pill. Contraceptive methods, such as IUDs and Fertility Awareness-Based Methods, are associated with considerable uncertainty among former oral contraceptive users, which aligns with findings from other studies [36, 37]. Education and counseling are vital in empowering individuals to make informed choices regarding their reproductive health. Contraceptive providers should deliver comprehensive information about all available contraceptive methods and address concerns and misconceptions to support patients in their decision-making process [38].

Another salient issue is the limited accessibility of sterilization as a long-term contraceptive option, particularly in young women. These barriers significantly impact contraceptive autonomy and agency. Our results are similar to those of other scholars [39–41]. However, there is a need for more in-depth research on voluntary female sterilization and structural and institutional barriers, especially for young individuals seeking contraception.

The need for alternative contraceptive options is underscored by recent studies, highlighting a shift in the way individuals approach reproductive health. Cetera et al. [42] emphasize that younger individuals prioritize safety and autonomy in their contraceptive choices, reflecting broader changes in attitudes toward reproductive health. Our analysis supports this, as interviewees expressed a desire for a method that combined the contraceptive safety of the pill with the noninvasiveness of short-term methods. Zettermark’s [43] work in Sweden further explores this issue, using reproductive justice frameworks to show how women seek methods that honor bodily autonomy and adapt to various life circumstances, preferences that mirror the participants’ emphasis on less invasive, user-centered solutions. This integration of the literature and findings creates a cohesive argument, demonstrating that our results are part of a larger conversation in the field.

The need for further research on the significance of equality in the development and provision of contraceptive methods is evident. This is evidenced by the paucity of literature addressing the contraceptive decisions of same-sex or queer couples. Future research could explore how traditional gender roles influence the selection of contraceptives in such partnerships.

Our study has several limitations. The participants’ responses could have been affected by their desire to appear socially acceptable. Although our sample size was adequate to generate insights into chaplains’ experiences, its limited scope prevented us from capturing a comprehensive range of religious or social backgrounds in Germany. Furthermore, we did not interview minors or non-German and non-English speakers, which potentially limits the applicability of our findings to these groups. Finally, our interview guide did not directly address cost-related themes, and participants did not mention financial considerations when discussing contraceptive decision-making. However, this may be an important factor in economically challenged individuals seeking contraception.

## Conclusions

Our study adds to critical research on the discontinuation of the contraceptive pill. We examined the factors influencing contraceptive choices after the discontinuation of oral contraceptives. Key considerations include efficacy, familiarity, preference for hormone-free options, biopsychological factors, shared contraceptive responsibility in partnerships, and the desire for safe, natural alternatives. The study also revealed challenges in contraception, particularly in providing IUDs for teenagers and voluntary sterilization for younger women, highlighting issues of contraceptive autonomy and agency. These findings underscore the need for comprehensive contraceptive counseling that addresses individual preferences, concerns, and experiences. Future research and policy efforts should focus on developing diverse, safe, and accessible contraceptive options that meet the varied needs and preferences of individuals across different life stages.

## Data Availability

The datasets generated and analyzed during the current study are not publicly available to protect the privacy of the interviewees. More detailed information on the data generation is available from JN upon request.

## Footnotes

1 We acknowledge that all individuals should have access to modern contraception. In this manuscript, we use the term ‘(former) pill users’ or ‘individual seeking contraception’, discussing it as a sexual and reproductive right. However, when referring to a specific study, we use the language chosen by the authors to describe the participants throughout their publications.

2 The symptothermal method is a fertility-awareness approach that involves observing changes in cervical mucus and recording daily basal body temperature to determine the fertile and infertile phases of the menstrual cycle [27].

3 All names are pseudonyms.
